# Risk Assessment of Adverse Birth Outcomes in Relation to Maternal Age

**DOI:** 10.1371/journal.pone.0114843

**Published:** 2014-12-10

**Authors:** Yi-Hao Weng, Chun-Yuh Yang, Ya-Wen Chiu

**Affiliations:** 1 Division of Neonatology, Department of Pediatrics, Chang Gung Memorial Hospital, Chang Gung University College of Medicine, Taipei, Taiwan; 2 Department of Public Health, Kaohsiung Medical University, Kaohsiung, Taiwan; 3 Master Program in Global Health and Development, College of Public Health and Nutrition, Taipei Medical University, Taipei, Taiwan; 4 Health Policy and Care Research Center, Taipei Medical University, Taipei, Taiwan; The Ohio State Unversity, United States of America

## Abstract

**Background:**

Although a number of studies have investigated correlations of maternal age with birth outcomes, an extensive assessment using age as a continuous variable is lacking. In the current study, we estimated age-specific risks of adverse birth outcomes in childbearing women.

**Method:**

National population-based data containing maternal and neonatal information were derived from the Health Promotion Administration, Taiwan. A composite adverse birth outcome was defined as at least anyone of stillbirth, preterm birth, low birth weight, macrosomia, neonatal death, congenital anomaly, and small for gestational age (SGA). Singletons were further analyzed for outcomes of live birth in relation to each year of maternal age. A log-binomial model was used to adjust for possible confounders of maternal and neonatal factors.

**Results:**

In total, 2,123,751 births between 2001 and 2010 were utilized in the analysis. The risk of a composite adverse birth outcome was significantly higher at extreme maternal ages. In specific, risks of stillbirth, neonatal death, preterm birth, congenital anomaly, and low birth weight were higher at the extremes of maternal age. Furthermore, risk of macrosomia rose proportionally with an increasing maternal age. In contrast, risk of SGA declined proportionally with an increasing maternal age. The log-binomial model showed greater risks at the maternal ages of <26 and > 30 years for a composite adverse birth outcome.

**Conclusions:**

Infants born to teenagers and women at advanced age possess greater risks for stillbirth, preterm birth, neonatal death, congenital anomaly, and low birth weight. Pregnancies at advanced age carry an additional risk for macrosomia, while teenage pregnancies carry an additional risk for SGA. The data suggest that the optimal maternal ages to minimize adverse birth outcomes are 26∼30 years.

## Background

There is a trend in increasing maternal age for childbearing worldwide. Changes in maternal age may have impacts on birth outcomes [1]–[5]. The etiologies of adverse birth outcomes are multifactorial and not completely understood yet. There are several indices of adverse birth outcomes, such as stillbirth, preterm birth, low birth weight, small for gestational age (SGA), macrosomia, neonatal death, and congenital anomaly. Stillbirth is one of the adverse birth outcomes of greatest concern. In addition, the birth weight and gestational age are important indicators of neonatal morbidity and mortality. An increasing number of publications have shown that pregnancies by teenagers and women of advance maternal age, defined as ≥35 years of age, are at greater risk for stillbirth, preterm birth, and low birth weight [1]–[22]. Researchers analyzed the relationship between maternal age and adverse birth outcomes by adjusting for maternal socio-economic status (such as prenatal care, marital status, residence, educational level, tobacco and alcohol consumption, and ethnicity) [1], [6]–[11], obstetric conditions (such as multiple pregnancy, parity, delivery mode, and pregnancy-related complications) and neonatal outcomes (such as stillbirth, gender, Apgar score, birth weight, and gestational age) [1], [2], [4], [6]–[16].

Some studies that examined the relationship between maternal age and adverse birth outcomes used data from regional samples or from a limited number of medical institutions [1], [3], [4], [10]–[12], [15]–[17]. Furthermore, most published studies categorized maternal age to analyze the association. An extensive assessment using age as a continuous variable is lacking. In the current study, we explored nationwide population-based data of over 2 million births by each year of maternal age to comprehensively analyze the adverse birth outcomes.

## Methods

The study protocol was approved by the Research Ethics Committee of the National Health Research Institutes in Taiwan. All records of participants were anonymized and de-identified prior to analysis. Targets of this retrospective population-based study were all births from 1 January 2001 to 31 December 2010 in Taiwan.

### Data resource

Maternal and neonatal data were derived from the Birth Notification System (BNS), a database established by the Health Promotion Administration, Ministry of Health and Welfare, Taiwan (Table 1). Medical organizations and midwives have to report all births to this system via an online reporting system within 7 days. If there are changes in the reported data, revision via the online reporting system is mandatory within 60 days. The birth registration data obtained from the BNS has been shown having good validity and reliability in birth outcomes [23], [24]. The Health Promotion Administration provided maternal and neonatal data and approved the use for this study.

**Table 1:** Definition of study covariates.

Covariate	Information from Birth Notification System
***Adverse birth outcomes***	
Stillbirth	death of a fetus at ≥20th weeks of gestation
neonatal death	death within 30 days of life
preterm birth (<37 weeks of gestation)	gestational age
low birth weight (<2500 g)	birth weight
macrosomia (≥4000 g)	birth weight
congenital anomaly	neonatal abnormality of chromosome and central nervous, craniofacial, cardiovascular, digestive, urogenital, skeletomuscular, and respiratory systems
SGA (birth weight below the 10th percentile for the gestational age)	gestational age, birth weight
***Maternal and neonatal confounders***	
maternal age (y)	maternal birthday
birth year (2001∼2010)	neonatal birthday
Taiwanese vs. non-Taiwanese	maternal ethnicity
male vs. female	gender
urban vs. suburban	birth region (city, county)
primipara vs. multipara	parity
pregnancy-related disorder	maternal anemia, diabetes, pregnancy-induced hypertension, toxemia
obstetric complication	maternal fever at delivery (>38°C), meconium in the amniotic fluid, premature rupture of membrane (>12 h), placental abruption, placenta previa, massive bleeding, seizure at delivery, precipitating delivery (<3 h), breech presentation/mal-presentation, cord prolapse, prolonged labor, dysfunctional labor, fetal distress, and complications of anesthesia
<7 vs. 7–10	Apgar score at 1 and 5 minutes
Cesarean section vs. vaginal delivery	delivery mode

### Adverse birth outcomes

Adverse birth outcomes were measured using the following 7 outcomes: stillbirth, preterm birth, neonatal death, congenital anomaly, low birth weight, macrosomia, and SGA (Table 1). A composite adverse birth outcome was defined as any of the above 7 adverse birth outcomes.

### Maternal age

Maternal age was defined as the age at delivery, which was calculated by subtracting the maternal birthday from the neonatal birthday. Women with a delivery age of <20 years old were classified as teenage mothers.

### Population for analyses

All births were included when estimating the stillbirth and composite adverse birth outcome. Otherwise, cases of stillbirths and multiple births were excluded when measuring the correlations of maternal age with preterm birth, low birth weight, neonatal death, macrosomia, congenital anomaly, SGA, and delivery mode.

### Statistical analyses

Statistical analyses were conducted using a commercially available program (SPSS 19.0 for Windows, SPSS., Chicago, IL, USA). Categorical variables were analyzed using a chi-squared test. Relative risk with 95% confidence intervals (CI) was expressed. For comparison between groups with quantitative variables, the null hypothesis that there was no difference between each group was tested by a one-way analysis of variance (ANOVA). Population attributable fraction (PAF) was estimated by the following formula:

PAF  =  [P(RR-1)]/[P(RR-1)+1]

P: proportion of composite adverse birth outcomes; RR: risk ratio

A log-binomial model (generalized linear model with a log link and a binomial distribution for the error term) was used to estimate the risk of adverse birth outcomes in relation to maternal age after adjusting for possible confounders of maternal and neonatal factors – including pregnancy-related disorders, birth region, parity, obstetric complications, ethnicity, birth year, sex at birth, congenital anomaly, neonatal death, Apgar score, delivery mode, gestational age, and birth weight. All covariates are defined in Table 1. Confounders were not used as covariates when they were dependents. These confounders were used for the binomial analysis because they have been documented to be associated with maternal age [25]–[28]. Significance was defined as *p*<0.05. Absolute risk difference and 95% CI were adjusted for the control variables.

## Results

Data on 2,123,781 births were collected in Taiwan from 2001 to 2010. We excluded 30 births with incomplete data, leaving 2,123,751 births for the analysis. There were 20,489 stillbirths (0.96%). Among 2,045,748 singleton live births, 62,839 neonates (3.07%) were born to teenage mothers and 243,624 neonates (11.91%) were born to mothers of ≥35 years of age. Socio-demographic data of the study population are summarized in Table 2. The number of births declined year by year from 250,079 births in 2001 to 168,504 births in 2010. Furthermore, there was a significant trend in the increasing age of childbearing women from 28.08 years in 2001 to 30.64 years in 2010 (*p*<0.001). Seven adverse birth outcomes are illustrated year by year in the S1 Figure.

**Table 2:** Socio-demographic data of study population.

Maternal age (year)	Number (%)	Maternal ethnicity (Taiwanese, %)	Residency (urban, %)
≤14	657 (0.03)	99.7	43.3
15	1898 (0.09)	99.2	34.6
16	4792 (0.23)	99.5	31.8
17	8927 (0.42)	99.3	29.7
18	15725 (0.74)	90.6	29.5
19	31892 (1.50)	76.5	30.3
20	46186 (2.17)	71.4	31.4
21	61888 (2.91)	70.2	31.6
22	76592 (3.61)	72.0	32.8
23	92300 (4.35)	75.7	34.2
24	107968 (5.08)	79.3	35.7
25	124073 (5.84)	82.7	37.2
26	141767 (6.68)	86.1	39.1
27	160788 (7.57)	88.9	41.5
28	173117 (8.15)	90.9	44.5
29	167574 (7.89)	92.1	47.1
30	164091 (7.73)	93.1	49.3
31	153504 (7.23)	93.7	51.8
32	136702 (6.44)	94.0	53.9
33	113629 (5.35)	94.2	55.3
34	91988 (4.33)	94.4	56.6
35	72484 (3.41)	94.3	57.3
36	55301 (2.60)	94.5	58.0
37	40545 (1.91)	94.7	58.7
38	28781 (1.36)	94.9	58.0
39	19940 (0.94)	94.7	58.9
40	13316 (0.63)	94.1	59.4
41	8096 (0.38)	94.4	59.6
42	4478 (0.21)	94.2	57.5
43	2470 (0.12)	94.7	57.4
≥44	2282 (0.11)	92.5	58.7

### Relative risk of adverse birth outcomes in relation to maternal age by univariate analysis

The risk of a composite adverse birth outcome was highest at ages of ≤14 years, then declined to an age of 27 years, and then steadily increased to ages of ≥44 years (Table 3). In addition, risks of composite adverse birth outcomes were significantly higher at maternal ages of ≤26 and ≥30 years when compared with maternal age of 27 years. Furthermore, PAFs were 0.94% (95% CI  = 0.88%–1.00%) and 2.35% (95% CI  = 2.23%–2.47%) for teenagers and women of advanced age, respectively. Risk of the following 7 adverse birth outcomes in relation to maternal age by univariate analysis is summarized in Table 4.

**Table 3:** Relative risk of composite adverse birth outcome in relation to maternal age among 2,123,751 births in 2001∼2010.

	composite adverse birth outcome		
		Risk (95% CI)	
Maternal age (y)	%	Crude	Adjusted
≤14	42.9	2.50 (2.28–2.73)*	3.84 (3.11–4.74)*
15	33.9	1.97 (1.85–2.10)*	2.53 (2.22–2.88)*
16	29.1	1.69 (1.62–1.77)*	2.03 (1.86–2.21)*
17	26.9	1.56 (1.51–1.62)*	1.87 (1.75–2.00)*
18	24.2	1.41 (1.37–1.45)*	1.55 (1.47–1.64)*
19	21.9	1.27 (1.24–1.30)*	1.38 (1.32–1.43)*
20	20.3	1.18 (1.16–1.20)*	1.25 (1.20–1.29)*
21	19.5	1.14 (1.11–1.16)*	1.19 (1.15–1.23)*
22	18.8	1.09 (1.07–1.11)*	1.11 (1.08–1.14)*
23	18.4	1.07 (1.05–1.09)*	1.07 (1.04–1.10)*
24	17.9	1.04 (1.02–1.06)*	1.03 (1.01–1.06)*
25	17.7	1.03 (1.01–1.04)*	1.03 (1.01–1.06)*
26	17.5	1.02 (1.00–1.04)*	1.02 (1.00–1.05)
27	17.2	1	1
28	17.3	1.01 (0.99–1.02)	1.00 (0.98–1.02)
29	17.4	1.02 (1.00–1.03)	1.01 (0.99–1.03)
30	17.5	1.02 (1.00–1.04)*	1.01 (0.99–1.04)
31	17.8	1.04 (1.02–1.06)*	1.03 (1.00–1.05)*
32	18.2	1.07 (1.05–1.09)*	1.06 (1.04–1.09)*
33	18.6	1.10 (1.08–1.12)*	1.08 (1.05–1.10)*
34	19.3	1.15 (1.13–1.18)*	1.14 (1.11–1.17)*
35	20.2	1.22 (1.19–1.24)*	1.19 (1.16–1.23)*
36	21.1	1.29 (1.26–1.32)*	1.25 (1.22–1.29)*
37	22.0	1.36 (1.32–1.40)*	1.31 (1.27–1.36)*
38	22.8	1.43 (1.38–1.47)*	1.39 (1.34–1.44)*
39	23.3	1.46 (1.41–1.51)*	1.39 (1.33–1.45)*
40	23.7	1.49 (1.43–1.56)*	1.41 (1.34–1.49)*
41	25.8	1.68 (1.59–1.77)*	1.59 (1.49–1.69)*
42	25.8	1.67 (1.56–1.79)*	1.68 (1.55–1.82)*
43	28.2	1.89 (1.73–2.07)*	1.86 (1.68–2.07)*
≥44	32.8	2.35 (2.15–2.57)*	2.29 (2.06–2.53)*

* p<0.05. Covariates: pregnancy-related disorders, obstetric complications, ethnicity, birth region, birth year, parity, sex at birth, Apgar score, and delivery mode.

**Table 4:** Relative risk of 7 adverse birth outcomes in relation to maternal age by univariate analysis.

	risk (95% CI) of adverse birth outcome						
Maternal age (y)	*Stillbirth* †	*Preterm birth* ‡	*Low birth weight* ‡	*Neonatal death* ‡	*Macrosomia* ‡	*Congenital anomaly* ‡	*SGA* ‡
≤14	12.6 (9.89–16.0)*	3.79 (3.27–4.38)*	3.97 (3.39–4.66)*	7.44 (4.22–13.13)*	0.25 (0.08–0.78)*	1.52 (1.10–2.06)*	1.88 (1.50–2.35)*
15	6.57 (5.36–8.05)*	2.67 (2.41–2.97)*	2.74 (2.45–3.08)*	3.08 (1.84–5.14)*	0.33 (0.19–0.58)*	1.51 (1.18–1.91)*	1.78 (1.56–2.04)*
16	4.14 (3.50–4.89)*	2.03 (1.88–2.20)*	2.36 (2.18–2.56)*	2.47 (1.72–3.55)*	0.39 (0.28–0.54)*	1.41 (1.01–1.97)*	1.83 (1.68–1.99)*
17	2.43 (2.06–2.85)*	1.78 (1.68–1.90)*	2.32 (2.18–2.46)*	2.33 (1.76–3.08)*	0.35 (0.27–0.45)*	1.56 (1.23–1.97)*	1.91 (1.80–2.03)*
18	1.92 (1.67–2.21)*	1.53 (1.45–1.61)*	2.12 (1.83–2.45)*	1.96 (1.55–2.49)*	0.47 (0.39–0.55)*	1.28 (1.05–1.56)*	1.73 (1.65–1.82)*
19	1.43 (1.27–1.61)*	1.30 (1.24–1.35)*	1.62 (1.55–1.69)*	1.67 (1.39–2.02)*	0.53 (0.47–0.59)*	1.22 (1.05–1.41)*	1.60 (1.54–1.67)*
20	1.19 (1.07–1.33)*	1.19 (1.15–1.24)*	1.44 (1.39–1.50)*	1.26 (1.05–1.51)*	0.57 (0.52–0.62)*	1.16 (1.02–1.32)*	1.48 (1.43–1.54)*
21	1.16 (1.05–1.29)*	1.13 (1.09–1.17)*	1.38 (1.33–1.43)*	1.42 (1.21–1.67)*	0.62 (0.58–0.68)*	1.14 (1.01–1.28)*	1.40 (1.35–1.44)*
22	0.99 (0.89–1.09)	1.09 (1.06–1.13)*	1.27 (1.23–1.31)*	1.29 (1.10–1.50)*	0.68 (0.64–0.73)*	1.07 (0.96–1.20)	1.31 (1.27–1.35)*
23	1.01 (0.92–1.11)	1.05 (1.02–1.08)*	1.17 (1.13–1.21)*	1.13 (0.97–1.32)	0.79 (0.75–0.84)*	1.02 (0.91–1.14)	1.25 (1.22–1.29)*
24	1.03 (0.94–1.12)	1.03 (1.00–1.07)*	1.12 (1.08–1.16)*	1.19 (1.03–1.37)*	0.82 (0.78–0.87)*	1.02 (0.92–1.13)	1.15 (1.12–1.18)*
25	1.00 (0.92–1.09)	1.02 (0.99–1.05)	1.08 (1.05–1.12)*	1.08 (0.94–1.25)	0.93 (0.88–0.98)*	0.94 (0.85–1.04)	1.11 (1.08–1.15)*
26	1.00 (0.92–1.08)	1.00 (0.97–1.03)	1.05 (1.02–1.09)*	1.00 (0.87–1.15)	0.92 (0.88–0.97)*	1.02 (0.93–1.12)	1.07 (1.04–1.10)*
27	1	1	1	1	1	1	1
28	1.04 (0.96–1.12)	1.00 (0.97–1.02)	1.01 (0.98–1.03)	0.92 (0.81–1.06)	1.05 (1.00–1.10)	0.99 (0.91–1.09)	0.97 (0.94–1.00)*
29	1.08 (1.00–1.16)	1.05 (1.02–1.08)*	1.02 (0.99–1.05)	0.93 (0.81–1.07)	1.08 (1.02–1.13)*	0.97 (0.88–1.06)	0.92 (0.90–0.95)*
30	1.09 (1.00–1.17)*	1.05 (1.02–1.08)*	1.00 (0.97–1.03)	0.94 (0.82–1.08)	1.14 (1.09–1.20)*	0.99 (0.90–1.09)	0.89 (0.87–0.92)*
31	1.20 (1.11–1.30)*	1.08 (1.05–1.12)*	1.01 (0.98–1.05)	0.93 (0.81–1.07)	1.20 (1.14–1.26)*	1.05 (0.96–1.15)	0.87 (0.84–0.89)*
32	1.27 (1.17–1.37)*	1.12 (1.09–1.15)*	1.02 (0.98–1.05)	1.03 (0.89–1.18)	1.25 (1.19–1.32)*	1.12 (1.02–1.23)*	0.84 (0.82–0.87)*
33	1.29 (1.19–1.40)*	1.17 (1.13–1.20)*	1.04 (1.01–1.08)*	1.00 (0.86–1.16)	1.36 (1.29–1.43)*	1.08 (0.98–1.20)	0.81 (0.79–0.84)*
34	1.56 (1.43–1.69)*	1.23 (1.19–1.27)*	1.10 (1.06–1.14)*	0.99 (0.84–1.16)	1.41 (1.34–1.49)*	1.11 (1.00–1.24)	0.80 (0.77–0.83)*
35	1.73 (1.59–1.88)*	1.33 (1.28–1.37)*	1.13 (1.09–1.17)*	1.08 (0.91–1.28)	1.48 (1.40–1.56)*	1.19 (1.06–1.33)*	0.81 (0.78–0.84)*
36	1.92 (1.76–2.10)*	1.44 (1.39–1.50)*	1.21 (1.16–1.26)*	1.15 (0.96–1.37)	1.66 (1.57–1.76)*	1.27 (1.12–1.43)*	0.79 (0.76–0.82)*
37	2.09 (1.90–2.30)*	1.53 (1.47–1.59)*	1.26 (1.21–1.33)*	1.35 (1.12–1.64)*	1.76 (1.65–1.87)*	1.25 (1.09–1.44)*	0.83 (0.79–0.87)*
38	2.11 (1.89–2.35)*	1.67 (1.60–1.75)*	1.37 (1.31–1.45)*	1.30 (1.04–1.62)*	1.80 (1.68–1.93)*	1.31 (1.13–1.53)*	0.83 (0.78–0.87)*
39	2.73 (2.44–3.05)*	1.69 (1.60–1.78)*	1.48 (1.41–1.56)*	1.19 (0.91–1.56)	1.83 (1.68–1.99)*	1.35 (1.13–1.61)*	0.87 (0.82–0.93)*
40	2.86 (2.51–3.26)*	1.80 (1.69–1.91)*	1.51 (1.41–1.61)*	1.66 (1.26–2.19)*	2.05 (1.87–2.25)*	1.31 (1.06–1.63)*	0.80 (0.74–0.87)*
41	3.54 (3.05–4.10)*	2.14 (1.99–2.29)*	1.64 (1.50–1.78)*	1.78 (1.27–2.49)*	2.12 (1.89–2.38)*	1.67 (1.31–2.12)*	0.82 (0.74–0.91)*
42	3.74 (3.10–4.51)*	2.20 (2.00–2.40)*	1.75 (1.57–1.95)*	2.30 (1.73–3.05)*	2.01 (1.71–2.34)*	1.72 (1.26–2.36)*	0.86 (0.76–0.99)*
43	4.70 (3.76–5.87)*	2.41 (2.14–2.71)*	2.00 (1.74–2.29)*	2.04 (1.18–3.55)*	2.22 (1.82–2.71)*	1.69 (1.10–2.58)*	1.01 (0.84–1.19)
≥44	5.78 (4.68–7.14)*	2.62 (2.31–2.97)*	1.93 (1.83–2.03)*	2.65 (1.46–4.61)*	1.76 (1.39–2.21)*	1.59 (1.01–2.51)*	0.95 (0.92–0.97)*

Reference year of maternal age: 27.

* p<0.05;

† n = 2,123,751 births;

‡ n = 2,045,748 singleton live births.

#### 1. Stillbirth

The highest risk of stillbirths was for ages of ≤14 years, then declined to ages of 22∼29 years, and then steadily increased to ages of ≥44 years. In particular, the risk of stillbirths at ages of ≥44 years was significantly lower than that at ages ≤15 years (p<0.001). The univariate analysis showed that pregnant women aged younger than 22 years or older than 29 years carried a greater risk for stillbirths compared to those aged 27 years.

#### 2. Preterm birth

Pregnant women aged <25 years or > 28 years carried a greater risk for preterm birth compared to those aged 27 years. The risk of preterm births was highest at ages of ≤14 years, then declined to ages of 25∼28 years, and then steadily increased to ages of ≥44 years.

#### 3. Low birth weight

Pregnant women aged <27 years or > 32 years carried a greater risk for having a low birth weight infant compared to those aged 27 years. The risk of low birth weight neonates was highest at ages of ≤14 years, which declined to ages of 27∼32 years, and then increased to an age of 43 years and older.

#### 4. Neonatal death

Risk of neonatal death was significantly higher at the extremes of maternal age. The risk of neonatal death was highest at ages of ≤14 years, which declined to an age of 28 years, and then increased to ages of ≥44 years.

#### 5. Macrosomia

There was a significant correlation of macrosomia with maternal age (p<0.001): risk of macrosomia was significantly lower at younger age and higher at older age. The lowest and highest risks of macrosomia were at ages of ≤14 and 43 years, respectively.

#### 6. Congenital anomaly

Congenital anomaly was more common in stillbirths than live births (data not shown). Risk of congenital anomaly was significantly higher at extreme maternal ages.

#### 7. SGA

There was a significant correlation of SGA with maternal age (p<0.001): risk of SGA was higher at younger age and lower at older age. The highest and lowest risks of SGA were at ages of ≤14 and 36 years, respectively.

### Delivery mode

In total, 686,491 births were by Cesarean section (CS) (33.56%). There was a significant correlation of delivery mode with maternal age (*p*<0.001): risk of CS was significantly lower at younger age and higher at older age (data not shown).

### Risk assessment of adverse birth outcomes in relation to maternal age by log-binomial model

The maternal age of 27 years was used as a reference since this age had the lowest risk for having a composite adverse birth outcome (Tables 3 & 5). The data showed that women aged <26 and > 30 years possessed a significantly higher risk of having a composite adverse birth outcome. Women aged ≤14 years carried the greatest risk for having a composite adverse birth outcome. Then the risks gradually declined with a rise in age to 26 years. The risk of having a composite adverse birth outcome appeared leveled at 26 ∼30 years of age. After the age of 30 years, the risk for having a composite adverse birth outcome increased year by year to ages of ≥44 years.

**Table 5:** Absolute risk difference of adverse birth outcomes in relation to maternal age by log-binomial model.

	absolute risk difference (95% CI)			
Maternal age (y)	*Composite* †	*Stillbirth* †	*Preterm birth* ‡	*Low birth weight* ‡
≤14	1.35 (1.14–1.56)*	2.64 (2.38–2.91)*	1.58 (1.32–1.84)*	1.58 (1.31–1.86)*
15	0.93 (0.80–1.06)*	1.96 (1.75–2.18)*	1.16 (0.99–1.33)*	1.09 (0.90–1.27)*
16	0.71 (0.62–0.79)*	1.44 (1.26–1.61)*	0.78 (0.66–0.90)*	0.90 (0.78–1.03)*
17	0.63 (0.56–0.69)*	0.90 (0.73–1.06)*	0.65 (0.56–0.75)*	0.94 (0.85–1.04)*
18	0.44 (0.39–0.49)*	0.66 (0.52–0.81)*	0.49 (0.41–0.57)*	0.75 (0.67–0.83)*
19	0.32 (0.28–0.36)*	0.38 (0.26–0.50)*	0.30 (0.23–0.36)*	0.53 (0.47–0.59)*
20	0.22 (0.18–0.25)*	0.17 (0.06–0.29)*	0.21 (0.15–0.26)*	0.39 (0.34–0.45)*
21	0.17 (0.14–0.20)*	0.17 (0.06–0.27)*	0.13 (0.08–0.18)*	0.34 (0.29–0.39)*
22	0.10 (0.07–0.13)*	0.00 (−0.10–0.10)	0.07 (0.02–0.12)*	0.22 (0.17–0.26)*
23	0.07 (0.04–0.10)*	0.01 (−0.09–0.10)	0.03 (−0.02–0.07)	0.15 (0.11–0.20)*
24	0.03 (0.01–0.06)*	0.02 (−0.06–0.11)	0.03 (−0.01–0.07)	0.12 (0.07–0.16)*
25	0.03 (0.01–0.06)*	0.01 (−0.08–0.09)	0.01 (−0.03–0.05)	0.09 (0.05–0.13)*
26	0.02 (−0.00–0.05)	0.00 (−0.08–0.09)	0.00 (−0.03–0.04)	0.07 (0.03–0.11)*
27	0	0	0	0
28	0.00 (−0.02–0.02)	0.04 (−0.03–0.12)	0.01 (−0.03–0.04)	0.02 (−0.02–0.06)
29	0.01 (−0.01–0.03)	0.08 (0.00–0.16)*	0.06 (0.02–0.09)*	0.02 (−0.02–0.06)
30	0.01 (−0.01–0.04)	0.08 (0.00–0.16)*	0.04 (0.01–0.08)*	0.02 (−0.02–0.06)
31	0.03 (0.01–0.05)*	0.18 (0.10–0.26)*	0.06 (0.03–0.10)*	0.01 (−0.03–0.05)
32	0.06 (0.04–0.08)*	0.24 (0.16–0.32)*	0.11 (0.08–0.15)*	0.03 (−0.01–0.07)
33	0.07 (0.05–0.10)*	0.26 (0.18–0.35)*	0.14 (0.10–0.17)*	0.04 (−0.00–0.08)
34	0.13 (0.10–0.15)*	0.45 (0.37–0.53)*	0.19 (0.15–0.23)*	0.10 (0.06–0.15)*
35	0.18 (0.15–0.20)*	0.55 (0.46–0.64)*	0.27 (0.23–0.31)*	0.11 (0.07–0.16)*
36	0.23 (0.20–0.25)*	0.65 (0.56–0.74)*	0.34 (0.30–0.39)*	0.16 (0.11–0.21)*
37	0.27 (0.24–0.31)*	0.75 (0.65–0.84)*	0.39 (0.34–0.43)*	0.21 (0.15–0.27)*
38	0.33 (0.29–0.36)*	0.76 (0.66–0.87)*	0.48 (0.43–0.53)*	0.27 (0.21–0.33)*
39	0.33 (0.29–0.37)*	1.01 (0.90–1.13)*	0.49 (0.42–0.55)*	0.35 (0.28–0.42)*
40	0.35 (0.30–0.40)*	1.06 (0.93–1.19)*	0.52 (0.45–0.60)*	0.35 (0.27–0.44)*
41	0.46 (0.40–0.52)*	1.25 (1.09–1.40)*	0.71 (0.63–0.80)*	0.44 (0.33–0.54)*
42	0.52 (0.44–0.60)*	1.34 (1.15–1.53)*	0.77 (0.66–0.88)*	0.56 (0.43–0.69)*
43	0.62(0.52–0.73)*	1.56 (1.33–1.78)*	0.88 (0.74–1.01)*	0.65 (0.49–0.81)*
≥44	0.83 (0.72–0.93)*	1.77 (1.56–1.98)*	0.94 (0.80–1.08)*	0.73 (0.57–0.90)*

Reference year of maternal age: 27.

* p<0.05.

† n = 2,123,751 births;

‡ n = 2,045,748 singleton live births. Covariates: (1) composite adverse birth outcome (at least one of stillbirth, preterm birth, low birth weight, congenital anomaly, macrosomia, neonatal death, and SGA): pregnancy-related disorders, obstetric complications, ethnicity, birth region, birth year, parity, sex at birth, Apgar score, and delivery mode; (2) stillbirth: pregnancy-related disorders, obstetric complications, ethnicity, birth region, birth year, sex at birth, parity, congenital anomalies, neonatal death, Apgar score, delivery mode, gestational age, and birth weight; (3) preterm birth: pregnancy-related disorders, obstetric complications, ethnicity, birth region, birth year, sex at birth, parity, congenital anomalies, neonatal death, Apgar score, delivery mode, and birth weight; (4) low birth weight: pregnancy-related disorders, obstetric complications, ethnicity, birth region, birth year, sex at birth, parity, congenital anomalies, neonatal death, Apgar score, delivery mode, and gestational age.

In specific, the greatest risk for having a stillbirth was at maternal ages of ≤14 years. The risk then declined proportionally with an increase in age to 21 years. Furthermore, there was no significant difference in the risk of having a stillbirth among women aged 22∼26 and 28 years compared to those aged 27 years. Then the risk gradually increased from age 29 years to ages of ≥44 years.

In addition, the greatest risk for having a preterm birth was at ages of ≤14 years. The risk then declined proportionally with an increase in age to 22 years. Furthermore, there was no significant difference in the risk of having a preterm birth among women aged 23∼26 and 28 years compared to those aged 27 years. In addition, the risk gradually increased from age 29 years to ages of ≥44 years.

Similarly, the greatest risk for having a low birth weight was being aged ≤14 years. The risk then gradually declined to an age of 26 years. Furthermore, there was no significant difference in the risk of having a low birth weight among women aged 28∼33 years compared to those aged 27 years. In addition, the risk gradually increased from an age of 34 years to ages of ≥44 years.

Furthermore, SGA and macrosomia were significantly associated with maternal age. In addition, there were no significant differences in neonatal death or congenital anomaly in relation to maternal age after adjusting for the control variables (data not shown).

## Discussion

The current evaluation illustrates correlations of maternal age with the following 7 adverse birth outcomes – stillbirth, preterm birth, neonatal death, congenital anomaly, macrosomia, SGA, and low birth weight – for over 2 million deliveries. Although previous investigations established maternal age to be an important factor in relation to adverse birth outcomes, data were less extensive in terms of estimates of the risk. In most previous studies, maternal age was categorized or dichotomized for the analysis. Our study differs from those studies in that we classified each year of age as an age-specific variable to allow for a more-precise and -realistic analysis of associations between maternal age and adverse birth outcomes. In addition, we provide a wealth of information regarding the public health magnitude of the issue by showing the risk of composite outcomes. Furthermore, we adjusted for control variables by log-binomial model and excluded some population (ex. multiple births) to minimize possible bias. Findings of our study demonstrate that women bearing children early or late in life possess a greater likelihood for composite adverse birth outcomes – including stillbirth, preterm birth, neonatal death, congenital anomaly, and low birth weight. In addition, pregnant women at advanced age are more likely to have CS and macrosomic infants. Furthermore, teenage pregnancies carry additional risk for SGA. To our knowledge, this is the first survey to comprehensively analyze age-specific risks of adverse birth outcomes.

Our study identified specific maternal ages which are at lower risk for a number of adverse birth outcomes during 2001∼2010 in Taiwan. We verified optimal ages for having a live birth as 22∼28 years. These data are consistent with previous reports showing greater risk of stillbirth at the extremes of reproductive age [7], [8], [10], [12], [14], [20]. In addition, the younger the maternal age, the lower the risk of macrosomia. Furthermore, maternal ages >27 years had lower risks of SGA. The findings suggest that birth weight is associated with maternal age.

In this study period, optimal ages of childbearing women to minimize preterm birth were 23∼28 years. In addition, optimal maternal ages to avoid low birth weight were 27∼33 years. These findings are largely consistent with those of other relevant studies showing that both teenagers and women of advanced maternal age are at greater risk for having preterm birth [3], [9]–[11], [15]–[17], [22] and low birth weight [2]–[4], [9], [11], [15], [16]. Of particular interest is the fact that in our study, the optimal maternal age for preventing low birth weight was greater than that for preventing preterm birth. Although in most circumstances the birth weight is proportional to gestational age [29], our data imply that the mechanisms of preterm birth and low birth weight in relation to maternal age partially differ [30]. Further investigations are required to clarify the differences.

It is well acknowledged that teenage pregnancies are at increased risk for adverse birth outcomes [6], [9], [10], [14], [16], [31]. Consistent with other studies [9], [21], [32], our results show a steep rise in adverse birth outcomes among adolescent pregnancies. It is important to note that the risks of stillbirth, preterm birth, neonatal death, and low birth weight among teenagers, especially extremely young mothers (<16 years), are significantly higher than childbearing women at advanced age. Low socioeconomic status, inadequate prenatal care, and inadequate weight gain during pregnancy may aggravate the risk of adverse birth outcomes for teenage deliveries [9], [17]. To our knowledge, this is the first report showing that pregnant adolescents aged <16 years were at the greatest risk for adverse outcomes among all age groups. The data suggest that medical professionals need to take into consideration the increased likelihood of adverse birth outcomes with pregnant teenagers. Nevertheless, the PAF of teenage delivery was less than that of delivery at advanced age, which is consistent with the fact that the number of pregnancy at teenage is less than that at advanced age. Thus, the population burden of adverse neonatal complications is more heavily influenced by women at advanced age than by teenagers. However, the PAF at both teenage and advanced age were relatively low. Over 95% of adverse birth outcomes occurred with mothers at 20∼34 years of age. The data lead to the conclusion that the burden of adverse birth outcomes is largely due to deliveries at maternal ages of 20∼34 years, not with teenagers or women at advanced age.

The mechanisms underlying the increased risk for adverse outcomes with extremes of maternal age are uncertain. Effects may result from direct biological changes or environmental impacts [30], [31]. A failure of the uterine vasculature may play a role in changes with maternal age [33]. Among older mothers, possible mechanisms include a higher incidence of obstetrical problems, such as miscarriage, preeclampsia, chronic hypertension, abnormal placental conditions, infertility, and their related management [34], [35]. As for adolescent pregnancies, reduced placental nutritional transport might compromise outcomes [36]. In addition to biological immaturity, socioeconomic factors, such as inappropriate care, might contribute to adverse birth outcomes from teenage pregnancies [21], [29], [37]. Further studies are needed to investigate the underlying mechanisms.

Our study illustrated that the risk of CS was proportional to maternal age. This was probably because of an increasing propensity to request cesarean delivery with maternal age [38]. A growing number of publications have shown that CS was more common among childbearing women at advanced age [1], [4], [39], [40]. One could question whether CS may impact the other adverse birth outcomes. Nevertheless, our study used a generalized linear regression analysis to control for the possible effect of delivery mode.

Our study showed a proportional rise of macrosomia with an increase in maternal age. This finding is consistent with previous reports [28], [41]. A possible reason is a higher body-mass index among older women. Nevertheless, there was a decreasing trend of macrosomia over the study period. We speculate adequate prenatal care may have contributed to this reduction. Further studies are needed to verify the mechanism. In addition, our study showed a slight association of maternal age with neonatal death. However, we found that these correlations were largely attributable to gestational age and birth weight.

There are limitations to this study. First, our samples were Asian and therefore cannot represent other races [42]–[44]. Second, we were unable to take socio-economic factors into account, such as the educational level and smoking status of the pregnant women. Third, we included pregnancies for those women who delivered more than once during the study period. Further studies are needed to clarify the impact of parity on adverse birth outcomes [25]. Despite those limitations, there are two major strengths of this study. First, our study is a population-based cohort, using a large nationwide sample size to analyze the risk of adverse birth outcomes. The birth registration data used in this study were shown to have good validity and reliability in birth outcomes [23], [24]. Second, the current study is the first survey to extensively determine age-specific risks of adverse birth outcomes. In addition, there was no significant change in the prenatal care during the study period since the policy has been guided by the National Health Insurance Administration, Ministry of Health and Welfare.

Our analyses illustrate a clear trend in reduced and delayed childbearing, which might have been driven by educational and labor gains of women over the last few decades. Women of childbearing age are confronted with the dilemma of choosing education, career, or pregnancy. Policy makers should consider designing certain feasible interventions to reduce the delivery age in an attempt to minimize adverse birth outcomes.

## Conclusions

The current study has measured adverse birth outcomes in relation to maternal reproductive age ranging from teenage to advanced age in a nationwide population-based setting. Our results demonstrate extremes of maternal age carry greater risks of stillbirth, preterm birth, neonatal death, congenital anomaly, and low birth weight with maternal age. In particular, pregnancies at very young age (<16 years) are at the greatest risk. The most optimal maternal ages to prevent overall adverse birth outcomes are 26∼30 years. Specifically, optimal maternal ages to minimize stillbirth, preterm birth, and low birth weight are 22∼28, 23∼28 and 27∼33 years, respectively. Our data highlight an imperative need to devise interventions to reduce adverse birth outcomes for pregnancies of teenagers and women of advanced age.

## Supporting Information

S1 Figure **Rates of adverse birth outcomes among 2,123,751 births.**
(TIF)
