# Supplementary figures and images for: Risk Assessment of Adverse Birth Outcomes in Relation to Maternal Age
Source: PLoS One. 2014 Dec 10;9(12):e114843. doi: 10.1371/journal.pone.0114843 (PMC4262474; doi:10.1371/journal.pone.0114843)

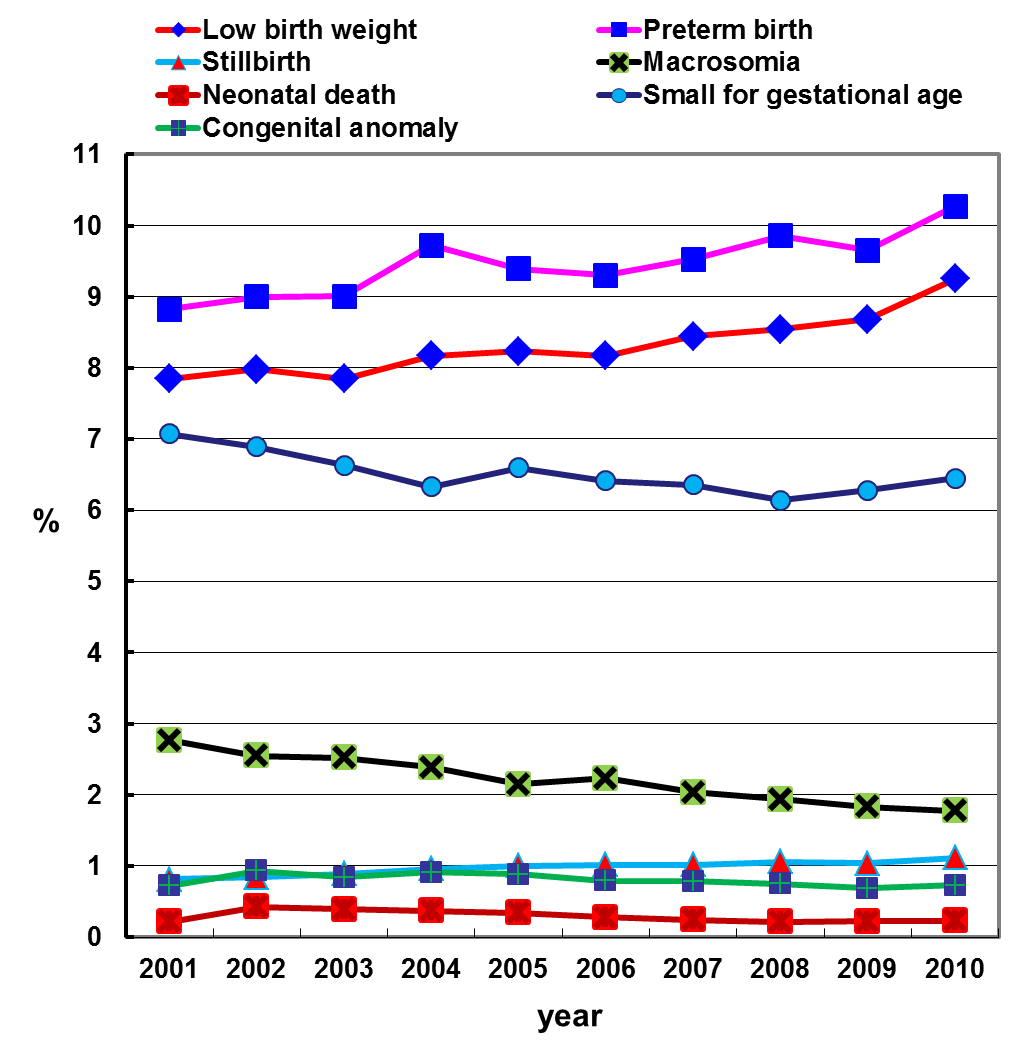

Supplement: S1 Figure — Rates of adverse birth outcomes among 2,123,751 births. (TIF) [file pone.0114843.s001.tif]
